# Intervention for physical activity in pregnant women with pre-pregnancy obesity or overweight based on the Fogg Behavior Model

**DOI:** 10.3389/fspor.2025.1594501

**Published:** 2025-09-18

**Authors:** Ying-li Song, Ji Zhang, Xiao Yang, Juan Wang, Xiang-zhi Wang

**Affiliations:** ^1^ Department of Obstetrics, Women and Infants Hospital of Zhengzhou, Zhengzhou, China; ^2^ School of Nursing, Henan University of Chinese Medicine, Zhengzhou, China; ^3^ Department of Musculoskeletal Pain Rehabilitation, The Fifth Affiliated Hospital of Zhengzhou University, Zhengzhou, China

**Keywords:** Fogg Behavior Model, physical activity, pre-pregnancy obesity or overweight, pregnant women, self-efficacy

## Abstract

**Objective:**

To investigate the impact of a physical activity intervention based on the Fogg Behavior Model on weight, gestational physical activity, self-efficacy for physical activity, and physical activity knowledge in pregnant women with pre-pregnancy obesity or overweight.

**Methods:**

A total of 132 pregnant women with pre-pregnancy obesity or overweight were divided into a control group and an intervention group, with 66 participants in each group. The control group received routine prenatal care, while the intervention group, based on the control group's regimen, received an intervention guided by the Fogg Behavior Model, focusing on the three key elements of motivation, ability, and prompts, until 36^+6^ weeks of gestation. Pre- and post-intervention evaluations were conducted for both groups, assessing maternal weight, gestational physical activity, self-efficacy for physical activity, and physical activity knowledge.

**Results:**

A total of 70 participants in the control group and 62 participants in the intervention group completed the intervention. After the intervention, the intervention group exhibited less weight gain in both the mid and late stages of pregnancy compared to the control group. The intervention group also had higher physical activity scores in both the mid and late stages of pregnancy. Additionally, self-efficacy for physical activity was higher in the intervention group during the mid-pregnancy period, and physical activity knowledge scores were higher in the intervention group during the late pregnancy period compared to the control group.

**Conclusion:**

Physical activity based on the Fogg Behavior Model is effective in reducing gestational weight gain and increasing physical activity during pregnancy.

## 1 Introduction

Physical activity refers to various bodily movements generated by skeletal muscle contractions that result in energy expenditure. According to the recommendations of the World Health Organization (WHO), pregnant women without medical contraindications should engage in at least 150 min of moderate-intensity physical activity per week (1). Pregnancy often leads to a significant reduction in physical activity among women (2), Studies conducted both domestically and internationally indicate that 84.2%–88.9% of pregnant women fail to meet the physical activity guidelines, demonstrating insufficient physical activity (3, 4). The WHO has identified physical inactivity as one of the major global public health challenges of the 21st century (5). Over the past two decades, the prevalence of overweight and obesity in China has risen rapidly (6). Among women of reproductive age, the overweight rate is 25.4%, and the obesity rate reaches 9.2% (7), Women who are overweight or obese before pregnancy face increased health risks during gestation. Research indicates that their risk of developing gestational diabetes mellitus (GDM) is four to nine times higher than that of women with normal weight, while the risk of preeclampsia increases by six to seven times. Additionally, the incidence of gestational hypertension and postpartum venous thromboembolism is four to five times higher. Moreover, they are at a significantly higher risk of cesarean section, miscarriage, stillbirth, preterm birth, and depression. The likelihood of delivering a macrosomic infant, congenital anomalies, and perinatal mortality also increases significantly (8). Regular physical exercise during pregnancy contributes to maintaining or improving overall health, supporting weight management, and reducing the risk of GDM in overweight or obese pregnant women. Furthermore, appropriate physical activity enhances psychological well-being and strengthens the ability to adapt to the physiological and psychological changes of pregnancy (9, 10).

Proposed by Stanford University Professor B.J. Fogg in 2009, the Fogg Behavior Model (FBM) is a theoretical framework designed to guide behavior change. It consists of three core elements: Motivation, Ability, and Prompt. The model posits that the successful execution of a target behavior depends on the synergistic interaction of these components. Motivation serves as the driving force behind behavior—higher motivation increases the likelihood of behavioral engagement. Ability reflects an individual's capacity to perform the behavior. when ability is high or the behavior itself is simple, habit formation becomes more attainable. Finally, the Prompt acts as the critical trigger for action—even with sufficient motivation and ability, the behavior may not occur without an effective cue. In recent years, the application of FBM has expanded significantly. Beyond its widespread use in product design (11–13), promotion of environmentally sustainable behaviors (14), and surveys of pension purchase intentions (15), FBM has gained increasing recognition in health management, particularly in chronic disease management. For instance, it has been employed to improve lifestyle habits in patients with osteoarthritis (16) and diabetes (17), as well as to enhance medication adherence among individuals with kidney disease (18). Globally, researchers have developed and implemented FBM-based behavior change interventions with notable success. These interventions have significantly increased HPV vaccination rates among adolescent females (19) and effectively promoted physical activity participation among community residents (20). Additionally, in the field of reproductive health, FBM has demonstrated positive outcomes in HIV prevention (21), contraceptive use promotion (22, 23), and management of childbirth-related fear. Militello et al. (24) further exemplified FBM's versatility by applying it to weight management in overweight and obese preschool-aged children in the United States, highlighting its potential in pediatric health interventions. In China, FBM has also been integrated into the design of pediatric rehabilitation training programs (24, 25). By leveraging its core mechanisms—enhancing motivation, building ability, and providing timely prompts—FBM equips parents with practical behavioral strategies while encouraging children to consistently practice and monitor new skills, thereby improving rehabilitation outcomes for children with disabilities. This program aims to promote healthy lifestyle adoption during pregnancy, offering a foundation for evidence-based prenatal exercise interventions. Moreover, it provides a framework for developing personalized intervention strategies tailored to this population.

## 2 Materials and methods

### 2.1 General information

A convenience sampling method was used to select pregnant women who registered for maternity care and underwent routine prenatal check-ups at the obstetric outpatient department of a tertiary maternal and child health hospital in Zhengzhou in February 2022 to April 2023. These women were followed up until 36^+6^ weeks of gestation. Eligible overweight or obese pregnant women were identified as study participants and enrolled between 5 and 12^+6^ weeks of gestation. They were randomly assigned to either the intervention group or the control group, with 73 participants in each group, using a random number table method. To ensure the scientific validity and comparability of the intervention effect evaluation based on FBM, baseline characteristics were systematically compared between the intervention and control groups prior to implementation. The assessed variables included demographic factors, pre-pregnancy weight status, health knowledge levels, physical activity self-efficacy, and physical activity compliance. This comparative analysis ensured equivalence between groups at baseline, minimizing potential confounding effects and strengthening the reliability of subsequent outcome assessments.

Inclusion criteria:

1. Naturally conceived singleton pregnancy, aged 18 years or older;
2. Undergoing regular routine prenatal check-ups;
3. No cognitive impairment, able to understand and respond to questions;
4. Willing to participate and providing signed informed consent;
5. Pre-pregnancy BMI >24 kg/m^2^.

Exclusion Criteria:

1. Contraindications to physical activity during pregnancy;
2. Pre-existing diabetes before pregnancy;
3. Cervical insufficiency;
4. Pre-existing medical conditions before pregnancy, including cardiovascular disease, pulmonary disease, hepatic disease, renal disease, hypertension, systemic lupus erythematosus, thyroid disorders, psychiatric disorders, or Urinary incontinence and pelvic organ prolapse;
5. Pregnant women currently taking medications such as metformin or corticosteroids.

Elimination Criteria:

1. Stillbirth or severe fetal malformations;
2. Incomplete pregnancy-related data.

### 2.2 Intervention methods

Pregnant women in the control group received routine prenatal care services, including risk assessment at the first prenatal examination and referral to an attending physician. They were advised to consult a nutrition clinic and encouraged to participate in health education sessions at the maternity school. Additionally, prenatal check-ups were conducted every four weeks before 28 weeks of gestation, every two weeks between 28 and 36 weeks, and weekly after 36 weeks until delivery. On the basis of routine health management, the intervention group received a physical activity program based on the FBM, which was developed and implemented as follows:

#### 2.2.1 Establishing an intervention team

Under the leadership of the principal researcher, a multidisciplinary intervention development team was formed, comprising two obstetricians, two outpatient obstetric nurses, one exercise specialist, and two research assistants. The researcher first presented the study's objectives, theoretical foundation, clinical practice guidelines, supporting literature, contextual factors, economic feasibility, and prior research findings to the team. Following this, the team engaged in collaborative discussions to systematically design stage-specific intervention strategies.

#### 2.2.2 Developing an intervention manual for pregnant women

Based on the research objectives and literature review, a health education manual on physical activity for pregnant women was developed, incorporating the FBM. The manual covers the definition, epidemiological characteristics, associated risks, and preventive measures for overweight and obese pregnant women. It also includes information on the benefits, types, appropriate timing, duration, intensity, and precautions of physical activity during pregnancy; the assessment and management of pregnancy-related discomforts; and the evaluation and intervention of prenatal depression and anxiety. Additionally, considering the physiological and psychological characteristics of pregnancy, an exercise guidance video tailored for pregnant women was produced. A physical activity log was also developed to facilitate the monitoring and management of their physical activity levels.

#### 2.2.3 Developing a prenatal exercise intervention program

Using the FBM as the theoretical framework, a prenatal exercise intervention program was developed through literature review, group discussions, and an assessment of participants' general characteristics and prenatal examination indicators. The motivation and ability for physical activity during pregnancy were analyzed, with fetal health, blood pressure, blood glucose levels, and gestational weight gain identified as cue signals for health-promoting behaviors. This approach helped define prenatal exercise goals, clarify exercise content, and determine effective intervention measures and strategies to promote behavioral changes in prenatal physical activity. Based on these findings, a preliminary draft of the FBM-based intervention program for physical activity in overweight or obese pregnant women was formulated.

To ensure the scientific rigor and feasibility of the program, two obstetricians with over 10 years of experience, one exercise specialist, and two outpatient obstetric nurses were invited for evaluation and review. The research team revised and refined the program based on expert feedback, ultimately finalizing a comprehensive intervention plan (see Table 1). Once established, the intervention program was compiled into a handbook, distributed to the multidisciplinary team members, and accompanied by a structured training session. The training covered the study objectives, program details, implementation procedures, and standardized intervention steps.

**Table 1:** Intervention on prenatal physical activity for overweight or obese pregnant women based on the Fogg Behavior Model.

Target behavior	Motivation	Ability	Trigger factors	Intervention content	Intervention measures and methods
Engaging in Regular Moderate-Intensity Physical Activity During Pregnancy	Enhancing Pregnant Women's Awareness of the Importance of Physical Activity During Pregnancy	(1) Pregnant women need to have the ability to reasonably plan their daily schedule to ensure sufficient time for moderate-intensity physical activity each day. (2) They should master the correct methods of performing moderate-intensity physical activities. (3) They should develop the ability to manage physical discomfort during pregnancy.	(1) Family support and encouragement. (2) Peer communication and support among pregnant women. (3) A safe and comfortable environment for physical activity.	(1) It is recommended that pregnant women engage in at least 150 min of moderate-intensity physical activity per week, with each session lasting no less than 30 min.	General Health Intervention: (1) Thematic Health Education Lectures – Conduct specialized health education sessions on prenatal health and physical activity. (2) Development and Distribution of the Prenatal Health Manual for Overweight or Obese Pregnant Women – A comprehensive, illustrated manual with detailed content to facilitate easy reading and learning for pregnant women. (3) Establishment of a WeChat Health Management Group – Organize pregnant women into an online health management group to regularly share prenatal exercise videos, health tips, and related information, enabling continuous learning and communication. (4) Initial On-Site Training at the Hospital – Conduct the first physical activity session as an in-person training at the hospital, guided by professional sports coaches and obstetric nurses to ensure correct exercise techniques.
Controlling Gestational Weight Gain	Stimulating Pregnant Women's Intrinsic Motivation – Emphasize the role of physical activity in improving childbirth outcomes and encourage pregnant women to actively participate in physical activities.	Ability to Self-Monitor Gestational Weight Gain	Daily Exercise Check-In Reminders via a Mini Program	Based on the pre-pregnancy BMI of pregnant women, reasonable gestational weight gain targets should be established. Regular weight monitoring should be conducted to ensure that gestational weight gain remains within a healthy range.	(1) Daily reminders via WeChat group for weight monitoring. (2) Weekly follow-up with pregnant women via telephone or WeChat to assess gestational weight gain, physical activity levels, address their questions, and adjust activity plans accordingly. (3) Encouragement of family member involvement in pregnant women's physical activities during pregnancy.
Benefits for the fetus	Enhancing maternal awareness of fetal health.	Having knowledge of the short-term and long-term impacts of maternal exercise during pregnancy on offspring.	Regular ultrasound monitoring of fetal weight, amniotic fluid volume, and related parameters during pregnancy.	Engaging in prenatal activities to promote healthy fetal development, reduce the risk of birth defects, and enhance offspring health outcomes after birth.	Follow-up: (1) Monitoring blood pressure, daily weight, and mid-pregnancy oral glucose tolerance test (OGTT) parameters. (2) Strengthening motivation, enhancing skills, and increasing reminders to sustain targeted behaviors. (3) Addressing questions raised by intervention participants. (4) Providing encouragement and rewards to participants who successfully establish healthy behaviors and achieve targeted behavioral goals.

#### 2.2.4 Implementing the intervention protocol

1. Evaluation: Assessing demographic characteristics, physiological parameters (e.g., pre-pregnancy BMI), motivation and capability for engaging in physical activity during pregnancy, cues to action, and pregnancy-related exercise promotion behaviors among intervention participants.
2. Intervention: According to the inclusion and exclusion criteria of this study, pregnant women with pre-pregnancy overweight or obesity and gestational ages within two weeks of each other were grouped into small cohorts. Each group consisted of 8–10 pregnant women. Participants in the intervention group received a physical activity intervention specifically designed for pregnant women with pre-pregnancy overweight or obesity, in addition to the standard care provided to the control group. The intervention spanned from 13 to 14^+6^ weeks of gestation through 36 weeks of gestation. Participants were instructed to engage in moderate-intensity physical activity at least five days per week. The physical activities included exercises derived from a systematic review (26), clinical practice guidelines (27), and expert recommendations from professional exercise coaches, comprising aerobic and resistance training. Aerobic exercises involved brisk walking as well as upper and lower limb stretching activities. Resistance training targeted major muscle groups, including the chest, back, shoulders, and upper and lower extremities. The intensity of physical activity was individualized based on perceived exertion levels measured by the Borg Rating Scale (target range: 12–14) (28) and the talk test criterion (ability to converse but not sing during exercise) (29). The intervention was guided by the FBM and comprised four core elements aimed at accumulating direct experiences, indirect learning, verbal encouragement, and physiological and emotional guidance. The intervention consisted of a total of 13 sessions, including one face-to-face group educational session and exercise clinic visit (direct experience), one online health education session focused on managing common pregnancy discomforts and emotional regulation (physiological and emotional guidance), and 11 online group meetings (indirect learning experiences). Additionally, daily reminders and motivational messages were provided to participants to encourage adherence to physical activity routines (verbal persuasion).
3. Follow-up: After completing one week of health intervention, follow-up continued until 36 weeks of pregnancy. Participants were required to attend in-person hospital follow-ups or receive follow-ups via WeChat/phone calls by the researchers at least once per week. During follow-ups, motivation was reinforced, capabilities were enhanced, and prompt factors were strengthened to maintain target behaviors.

### 2.3 Evaluation methods

Researchers conducted face-to-face paper-based questionnaire surveys to collect the following data at baseline, 24–27^+6^ weeks, and 35–36^+6^ weeks of gestation.

#### 2.3.1 Pregnancy physical activity questionnaire

This study adopted the Chinese version of the Pregnancy Physical Activity Questionnaire (PPAQ), translated and validated by Zhang Yan et al. (30) to assess pregnant women's physical activity levels. The questionnaire comprises 31 activities divided into four main categories: household activities (14 items), exercise activities (8 items), transportation-related activities (4 items), and occupational activities (5 items). Pregnant women were asked to specify the duration of each activity according to their individual circumstances, ranging from not performed at all to more than 3 h per day. Activity intensities were categorized into four levels based on Metabolic Equivalent of Energy (MET) values: sedentary (MET <1.5), low intensity (MET 1.5–3.0), moderate intensity (MET 3.0–6.0), and high intensity (MET >6.0). Additionally, activities were classified by type into caregiving at home, occupational activities, transportation, exercise, and inactivity. The Chinese version of the PPAQ demonstrated high content validity (0.940), excellent test-retest reliability (0.944), and a correlation coefficient of 0.768 when compared to accelerometer measures, indicating robust applicability within the Chinese pregnant population.

#### 2.3.2 Pregnancy exercise self-efficacy scale

In this study, the Pregnancy Exercise Self-efficacy Scale (P-ESES), translated and validated into Chinese by Yang Hongmei et al. (31) was employed to assess the self-efficacy for physical activity among pregnant women. The scale consists of 10 items, each rated on a five-point Likert scale ranging from 1 to 5, with total scores ranging from 10 to 50. Higher scores indicate greater self-efficacy in performing physical activities. Based on total scores, exercise self-efficacy is classified into three categories: low (10–20 points), moderate (21–40 points), and high (41–50 points). The scale has demonstrated good reliability among pregnant women, with a Cronbach's *α* coefficient of 0.80.

#### 2.3.3 Pregnancy physical activity knowledge questionnaire

The Pregnancy Physical Activity Knowledge Questionnaire developed by Yao Shanshan et al. (32) was used in this study to assess pregnant women's knowledge regarding physical activity during pregnancy. The questionnaire consists of seven items covering topics such as the effects of physical activity during pregnancy on maternal health, fetal health, and delivery mode, as well as suitable types of exercise, contraindications, and warning signs indicating that exercise should be discontinued. Each item provides three response options: “Aware,” “Uncertain,” and “Unaware.” A respondent who correctly answers five or more items is considered to have adequate knowledge regarding pregnancy-related physical activity, whereas a respondent correctly answering fewer than four items is regarded as having inadequate knowledge.

### 2.4 Statistical methods

Statistical description tests *χ*^2^, Wilcoxon rank sum test, *t*-test and generalized estimating equation analysis were conducted using SPSS 25.0 software, with the significance level *α* = 0.05.

## 3 Results

### 3.1 Comparison of basic characteristics between the two groups of pregnant women

The study retained 70 participants in the intervention group and 62 in the control group through completion. A summary of baseline characteristics is presented in Table 2. Regarding age and gestational weeks, the mean age of participants in the intervention group was 30.67 ± 3.71 years, compared to 30.76 ± 4.48 years in the control group. The mean gestational age was 11.81 ± 0.57 weeks and 11.89 ± 0.32 weeks in the intervention and control groups, respectively. No statistically significant differences were observed between the two groups (*p* > 0.05). In terms of parity, the intervention group included 39 primiparas and 31 multiparas, while the control group included 37 and 25, respectively, with no significant difference (*p* = 0.646), indicating good comparability in demographic characteristics.

**Table 2:** Comparison of basic characteristics between the Two groups of pregnant women ($\bar{x}\pm s$).

Factors	Intervention group (*n* = 70)	Control group (*n* = 62)	*t*/*χ*^2^ value	*p* value
Age (years)	30.67 ± 3.71	30.76 ± 4.48	−0.122¬	0.390
Mean gestational age (weeks)	11.81 ± 0.57	11.89 ± 0.32	−0.887¬	0.066
Maternal category			0.211­	0.646
Primipara (cases)	39	37		
Multipara (cases)	31	25		
Maternal weight				
Overweight pregnant women before pregnancy^a^ (cases)	50	42		
Pre-pregnancy weight (kg)	67.27 ± 4.57	67.94 ± 5.54	−0.636¬	0.124
Pre-pregnancy BMI (kg/m^2^)	25.51 ± 1.00	25.62 ± 1.15	−0.491¬	0.241
Obese Pregnant Women Before Pregnancy^b^ (cases)	20	20		
Pre-pregnancy weight (kg)	81.68 ± 9.34	80.88 ± 8.23	0.287¬	0.547
Pre-pregnancy BMI (kg/m^2^)	31.26 ± 2.87	30.07 ± 1.82	1.570¬	0.059
Knowledge Score			0.019­	0.890
Meeting the standard (cases)	2	3		
Not meeting the Standard (cases)	68	59		
Level of physical activity self-efficacy			0.842­	0.657
Lower (cases)	2	1		
Moderate (cases)	62	53		
Higher (cases)	6	8		
Meeting the physical activity standard			0.051­	0.821
Meeting the standard (cases)	42	36		
Not meeting the standard (cases)	28	26		

¬ Represents *t* value; ­Represents χ^2^ value;

^a^ Represents 24 kg/m^2^ ≤ BMI < 28 kg/m^2^.

^b^ Represents BMI ≥ 28 kg/m^2^.

For pre-pregnancy weight and body mass index (BMI), among women who were overweight before pregnancy, the mean weight in the intervention group was 67.27 ± 4.57 kg and BMI was 25.51 ± 1.00 kg/m^2^, compared to 67.94 ± 5.54 kg and 25.62 ± 1.15 kg/m^2^ in the control group. These differences were not statistically significant (*p* > 0.05). Among women classified as obese before pregnancy, the mean pre-pregnancy weight was 81.68 ± 9.34 kg and BMI was 31.26 ± 2.87 kg/m^2^ in the intervention group, while in the control group these were 80.88 ± 8.23 kg and 30.07 ± 1.82 kg/m^2^, respectively, with no statistically significant difference observed (*p* > 0.05); however, the BMI difference approached statistical significance (*p* = 0.059), suggesting slightly higher pre-pregnancy BMI among obese women in the intervention group.

In terms of health knowledge and physical activity-related indicators, the proportion of participants meeting the knowledge criteria was low in both groups, with two in the intervention group and three in the control group reaching the threshold, and no significant difference between groups (*p* = 0.890). Regarding physical activity self-efficacy, 68 participants (97.1%) in the intervention group and 61 participants (98.4%) in the control group demonstrated moderate or high levels, with no significant difference (*p* = 0.657). The proportion of participants meeting the physical activity recommendation was 60.0% in the intervention group and 58.1% in the control group, with no statistically significant difference (*p* = 0.821).

In summary, no significant differences were found between the intervention and control groups across baseline characteristics, indicating good comparability and establishing a sound foundation for evaluating the effects of the subsequent intervention.

### 3.2 Comparison of gestational weight gain between the two groups of pregnant women

To evaluate the impact of a physical activity intervention based on the Fogg Behavior Model (FBM) on gestational weight control among overweight or obese women prior to pregnancy, this study compared gestational weight gain between the intervention and control groups. The results are presented in Table 3. In the intervention group, 18 participants (25.7%) experienced insufficient weight gain, 24 (34.3%) achieved appropriate weight gain, and 28 (40.0%) had excessive weight gain. In the control group, the corresponding numbers were 6 (9.7%), 22 (35.5%), and 34 (54.8%), respectively. Statistical analysis indicated that the overall distribution of gestational weight gain differed significantly between the two groups (*χ*^2^ = 6.206, *p* = 0.045). The proportion of participants with excessive weight gain was notably lower in the intervention group compared to the control group, suggesting that the FBM-based physical activity intervention was effective in improving gestational weight control and reducing the risk of excessive weight gain among overweight or obese pregnant women.

**Table 3:** Comparison of gestational weight gain between the two groups of pregnant women.

Weight parameters	Intervention group (*n* = 70)	Control group (*n* = 62)	*χ*^2^ value	*p* value
Gestational weight gain			6.206	0.045
Inadequate gestational weight gain (cases)	18	6		
Adequate gestational weight gain (cases)	24	22		
Excessive gestational weight gain (cases)	28	34		

### 3.3 Comparison of pregnancy physical activity knowledge scores between groups

To assess the efficacy of a physical activity intervention grounded in FBM for enhancing knowledge acquisition among overweight or obese women in the preconception period, this study conducted a comparative analysis of physical activity knowledge scores between the intervention and control groups during both the second and third trimesters of pregnancy. The results are presented in Table 4. In the second trimester (24–27^+6^ weeks), 18 participants (25.7%) in the intervention group met the knowledge standard, while 52 (74.3%) did not. In the control group, only 8 participants (12.9%) met the standard, with 54 (87.1%) not reaching it. The difference between groups approached statistical significance (*χ*^2^ = 3.412, *p* = 0.065), suggesting an improvement in knowledge levels within the intervention group at this stage, although the result did not reach conventional significance. In the third trimester (35–36^+6^ weeks), the number of participants meeting the knowledge standard in the intervention group increased significantly to 29 (41.4%), while in the control group only 10 participants (16.1%) achieved this level. The number of participants not meeting the standard was 41 (58.6%) in the intervention group and 52 (83.9%) in the control group. The difference between groups was statistically significant (*χ*^2^ = 10.110, *p* = 0.001), indicating that by the later stage of the intervention, participants in the intervention group had significantly better knowledge regarding physical activity during pregnancy compared to those in the control group. These findings suggest that the FBM-based intervention had a positive effect on improving physical activity knowledge among overweight or obese pregnant women, with a more pronounced impact observed in the third trimester.

**Table 4:** Comparison of pregnancy physical activity knowledge scores between the two groups.

Knowledge scores	Intervention group (*n* = 70)	Control group (*n* = 62)	*χ*^2^ value	*p* value
Knowledge scores at 24–27^+6^ weeks			3.412	0.065
Achieved standard (cases)	18	8		
Did not achieve standard (cases)	52	54		
Knowledge scores at 35–36^+6^ weeks			10.110	0.001
Achieved standard (cases)	29	10		
Did not achieve standard (cases)	41	52		

### 3.4 Comparison of pregnancy physical activity self-efficacy scores between groups

To assess the impact of an FBM-based physical activity intervention on self-efficacy for physical activity among overweight or obese women prior to pregnancy, this study compared self-efficacy scores between the intervention and control groups during the second and third trimesters. Detailed results are presented in Table 5. In the second trimester (24–27^+6^ weeks), the number of participants in the intervention group with low, moderate, and high self-efficacy was 1 (1.4%), 49 (70.0%), and 20 (28.6%), respectively. In the control group, the corresponding figures were 3 (4.8%), 52 (83.9%), and 7 (11.3%). The distribution of self-efficacy levels between the two groups was statistically significant (*χ*^2^ = 6.889, *p* = 0.032), indicating that a higher proportion of participants in the intervention group had high self-efficacy. This suggests that the intervention had a positive effect on enhancing self-efficacy for physical activity during this stage of pregnancy. However, in the third trimester (35–36^+6^ weeks), the distribution of low, moderate, and high self-efficacy levels in the intervention group was 3 (4.3%), 55 (78.6%), and 12 (17.1%), respectively, compared to 3 (4.8%), 50 (80.6%), and 9 (14.5%) in the control group. No statistically significant difference was observed between the two groups (*χ*^2^ = 0.182, *p* = 0.913), indicating that as pregnancy progressed, self-efficacy levels in both groups tended to converge, and the intervention effect may have diminished in the later stage. In summary, the FBM-based intervention significantly improved self-efficacy for physical activity during the second trimester, but its sustained effect appeared to be limited in the third trimester. These findings highlight the need for maintaining the continuity of intervention and incorporating reinforcement strategies in the later stages of pregnancy.

**Table 5:** Comparison of pregnancy physical activity self-efficacy scores between the two groups.

Level of self-efficacy	Intervention group (*n* = 70)	Control group (*n* = 62)	*χ*^2^ value	*p* value
Self-efficacy at 24–27^+6^ weeks			6.889	0.032
Low level (cases)	1	3		
Moderate level (cases)	49	52		
High level (cases)	20	7		
Self-efficacy at 35–36^+6^ weeks			0.182	0.913
Low level (cases)	3	3		
Moderate level (cases)	55	50		
High level (cases)	12	9		

### 3.5 Comparison of physical activity compliance scores between the two groups during pregnancy

To further assess the efficacy of a physical activity intervention grounded in FBM for actual physical activity performance among overweight or obese women prior to pregnancy, this study compared physical activity compliance between the intervention and control groups during the second and third trimesters. The results are presented in Table 6. In the second trimester (24–27^+6^ weeks), 59 participants in the intervention group (84.3%) met the recommended level of physical activity, while 11 (15.7%) did not. In the control group, 41 participants (66.1%) met the standard, and 21 (33.9%) did not. Statistical analysis showed a significant difference between the groups (*χ*^2^ = 5.902, *p* = 0.015), indicating that the physical activity performance of the intervention group was superior to that of the control group at this stage, and that the intervention had a preliminary positive effect. In the third trimester (35–36^+6^ weeks), the number of participants in the intervention group meeting the physical activity standard increased to 63 (90.0%), with only 7 (10.0%) not meeting the standard. In the control group, 45 participants (72.6%) met the standard, while 17 (27.4%) did not. The difference between groups remained statistically significant (*χ*^2^ = 6.706, *p* = 0.010), suggesting that under continuous intervention, the physical activity compliance rate of the intervention group further improved, and the effect of the intervention remained significant in late pregnancy. These findings indicate that the FBM-based intervention significantly improved physical activity compliance in both the second and third trimesters among overweight or obese pregnant women. This demonstrates the model's practical value and effectiveness in promoting the translation of target behaviors into action.

**Table 6:** Comparison of achieving recommended physical activity levels between the two groups.

Physical activity compliance	Intervention group (*n* = 70)	Control group (*n* = 62)	*χ*^2^ value	*p* value
Compliance at 24–27^+6^ weeks				
Achieved recommended levels (cases)	59	41	5.902	0.015
Did not achieve recommended levels (cases)	11	21		
Compliance at 35–36^+6^ weeks				
Achieved standard (cases)	63	45	6.706	0.010
Did not achieve recommended standard (cases)	7	17		

## 4 Discussion

### 4.1 Intervention effects on gestational weight gain

This study found that the FBM-based intervention significantly reduced gestational weight gain among overweight and obese women prior to pregnancy, particularly in lowering the incidence of excessive weight gain. The rate of excessive gestational weight gain in the intervention group was 40.0%, significantly lower than that of the control group (54.8%), with a statistically significant difference (*p* = 0.045). This finding is consistent with results published in The Lancet (33), which highlight the strong association between pre-pregnancy overweight or obesity and excessive gestational weight gain. Poor weight control during pregnancy is known to significantly increase the risk of adverse pregnancy outcomes such as gestational diabetes, hypertensive disorders, macrosomia, and cesarean delivery. Therefore, promoting and sustaining healthy behaviors—particularly evidence-based physical activity patterns—remains a key focus in prenatal intervention research.

This result also aligns with findings from a systematic review on exercise interventions for overweight or obese pregnant women (34), which concluded that physical activity during pregnancy can help limit excessive gestational weight gain in this population. In a behavior theory-based intervention study, Flannery et al (35) reported that helping pregnant women set clear physical activity goals, enhance their execution capabilities, and receive continuous prompts can significantly reduce the risk of abnormal gestational weight gain. The FBM, which is structured around the three elements of motivation, ability, and prompt, effectively integrates the core mechanisms of behavior change and facilitates the achievement of target health behaviors. By incorporating the FBM framework into prenatal physical activity guidance, the present study demonstrates its suitability for high-risk pregnant populations.

Notably, unlike traditional interventions that primarily emphasize cognitive education, FBM places greater emphasis on the practical feasibility and sustainability of behavior pathways. For example, Militello et al. (24) applied the FBM in an intervention targeting overweight children and found that capacity building and real-time prompts were especially crucial for maintaining behavioral adherence. In the current study, the use of staged follow-up and reminder mechanisms helped participants maintain a high level of behavioral execution throughout pregnancy, thereby improving weight management outcomes.

Furthermore, most domestic studies remain focused on the dissemination of exercise knowledge (5), with intervention strategies that are relatively simple and lacking in structured design informed by behavioral models. This study introduced innovation in theoretical foundation, intervention implementation, and outcome evaluation, demonstrating that FBM has strong potential for cross-population and cross-context application. It is especially suitable for pregnancy-related health management scenarios that require sustained behavioral adjustment.

In conclusion, the FBM-based intervention strategy effectively promotes appropriate gestational weight gain and offers important practical value. Future research should explore the model's adaptability across diverse cultural contexts and its long-term effectiveness. Additionally, integrating digital health tools may further facilitate personalized behavioral interventions, thereby improving pregnancy health management for overweight and obese women.

### 4.2 Intervention effects on pregnant women's knowledge regarding physical activity

This study implemented a physical activity intervention for overweight or obese women prior to pregnancy based on three core dimensions of FBM: motivation enhancement, ability building, and prompt guidance. The results indicated that the systematic intervention under the FBM framework not only improved participants' cognitive knowledge about physical activity but also facilitated the internalization of that knowledge into actual health behaviors.

Previous research has confirmed that pregnant women's level of knowledge about physical activity significantly influences their behavioral adoption and long-term adherence. Katherine et al. (36) pointed out that when pregnant women possess sufficient knowledge about physical activity, they are more capable of assessing safety thresholds and thus more confident in engaging in physical activity interventions. However, traditional interventions often rely on one-time education sessions or passive dissemination of information, lacking continuity and interactivity, which limits the transformation of knowledge into practice. In contrast, FBM emphasizes a behavior formation pathway based on “motivation–ability–prompt” and addresses these limitations by incorporating regular interviews, group-based interactions, behavioral cues, and ability-enhancing strategies. In this study, the rate of knowledge attainment in the intervention group increased to 41.4% by the third trimester, significantly higher than the 16.1% observed in the control group, highlighting the model's advantage in behavior-oriented cognitive interventions.

Moreover, studies have shown that pregnant women's knowledge of health is closely associated with antenatal compliance and maternal–infant outcomes. In a community-based study in China, Wang et al. (37) found that women who received structured prenatal health education had better outcomes in weight control, delivery method selection, and breastfeeding rates compared to those who received routine education. Their study emphasized that contextualization and interactivity in knowledge delivery are key to improving intervention outcomes. Similarly, the intervention in the present study employed modular education, regular health content dissemination via WeChat groups, and personalized Q&A support, which together contributed to the sustainability and individualization of knowledge acquisition, reinforcing both memory retention and behavioral identification among participants.

In contrast to international practices, domestic scholars such as Liu Xiangmao et al. (5) have noted that despite initial progress in promoting the Guidelines for Physical Activity During Pregnancy, challenges remain in terms of the depth of knowledge dissemination and the effectiveness of knowledge-to-action translation. Embedding FBM into the logic of intervention design may help overcome these implementation gaps, especially the common “easy to understand but hard to apply” dilemma in health education.

In conclusion, this study confirmed that FBM-guided intervention strategies significantly improved physical activity knowledge among overweight or obese pregnant women, with particularly pronounced effects in the third trimester. The practical value of this model lies in its strong operability and clearly defined behavior transformation pathways. It is well suited for dual interventions targeting both knowledge and behavior in high-risk pregnancy populations. Future applications may extend to primary care settings or digital health platforms to promote more precise and intelligent prenatal health education.

### 4.3 Intervention effects on pregnant women's physical activity self-efficacy

In this study, the physical activity self-efficacy of pregnant women in the intervention group was significantly higher than in the control group during the second trimester (*p* = 0.032), however, this difference was no longer statistically significant by the third trimester. Before the intervention, most participants had moderate levels of physical activity self-efficacy. Post-intervention results indicated that the intervention significantly improved physical activity self-efficacy during the second trimester, particularly increasing the likelihood of achieving higher self-efficacy levels.

This finding is consistent with the objectives of the present study and aligns with the predictions of self-efficacy theory (38). However, it is worth noting that in the later stage of the intervention, self-efficacy levels in both groups began to converge, and the between-group difference was no longer statistically significant. This outcome may be attributed to two main factors: first, the increased physical burden in the third trimester may have led to subjective barriers such as mobility limitations and heightened fatigue, thereby reducing participants' confidence in engaging in physical activity; second, the frequency and content of the intervention may not have been sufficiently adjusted to meet the evolving needs of participants in late pregnancy, suggesting that late-stage interventions should place greater emphasis on dynamic feedback and individual differences.

Nicola et al. (39) noted that single-phase interventions often exhibit a “peak–decline” pattern in behavior change, and that sustained behavioral support combined with personalized reinforcement strategies is essential for maintaining intervention effectiveness. In light of this, the present study suggests that future practice should consider integrating digital tools—such as WeChat-based reminders, mobile app check-ins, and real-time exercise feedback mechanisms—to extend motivation-enhancing and ability-building strategies. Such approaches may help sustain and even enhance self-efficacy during the third trimester.

Furthermore, the present study incorporated the FBM framework into the design of prenatal education and physical activity promotion, offering a replicable theoretical model for interventions targeting overweight and obese pregnant women in China. Compared to traditional didactic education approaches, FBM-based interventions demonstrated stronger behavioral engagement and sustainability in improving self-efficacy, highlighting the model's practical value and scalability—particularly in community-based maternal health management and digital health integration.

In summary, the FBM-guided intervention strategy significantly enhanced self-efficacy for physical activity during the second trimester, effectively increasing participants' engagement and motivation. However, to sustain these benefits into late pregnancy, future interventions should be further refined to accommodate the behavioral characteristics and support needs specific to the later stages of gestation, thereby achieving continuous enhancement of self-efficacy and long-term behavioral maintenance.

### 4.4 Intervention effects on achieving recommended levels of physical activity

The study found that the intervention significantly increased moderate-intensity physical activity energy expenditure, duration of moderate-intensity activities, and the proportion of pregnant women meeting recommended physical activity levels, consistent with findings from Wang et al. (40). Post-intervention, the compliance rate for recommended physical activity levels in the intervention group (84.3%, 59/70) was significantly higher than in the control group (66.1%, 41/62), and this gap widened further in late pregnancy (90.0%, 63/70 vs. 72.6%, 45/62). These findings demonstrate the effectiveness of the FBM-based intervention in facilitating adherence to recommended physical activity levels among pregnant women. By simplifying behavioral goals, providing immediate feedback, and enhancing social support, the intervention effectively incorporated physical activities into participants' daily routines. Such behavioral changes closely reflect the core principles of FBM—lowering barriers, enhancing motivation, and providing triggers to encourage sustained behaviors.

However, the control group's increased physical activity over time primarily involved household and sedentary activities, while their vigorous and exercise-specific activities decreased. Conversely, the intervention group increased their moderate-to-vigorous physical activities and structured exercise participation. These results align with findings by Nobles et al. (41) indicating prenatal exercise interventions can enhance moderate-to-vigorous physical activity and exercise engagement. However, due to the influence of traditional cultural norms, cognitive misconceptions, and insufficient motivation, pregnant women who are overweight or obese prior to pregnancy often exhibit lower willingness to engage in physical activity and poorer behavioral adherence, resulting in generally low rates of physical activity compliance (42). This study introduced FBM and systematically designed an intervention pathway focusing on three core components—motivation enhancement, ability improvement, and prompt reinforcement—which effectively addressed the aforementioned barriers.

From the perspective of intervention mechanisms, the strength of FBM lies not only in its emphasis on the “three elements” of behavior execution but also in its ability to simplify behavioral pathways and reduce execution thresholds, thereby facilitating the initiation and maintenance of behaviors (43). In this study, participants in the intervention group engaged in personalized goal setting, used WeChat-based check-in reminders, and received peer support. These strategies improved the accessibility of physical activity, stimulated intrinsic motivation, and enhanced the initiative and sustainability of behavioral engagement in daily life.

In summary, the FBM-based intervention model significantly improved physical activity compliance during both the second and third trimesters among overweight or obese pregnant women, demonstrating strong operability and potential for sustained behavioral change. This model holds considerable promise for broader application in the management of high-risk pregnancies, the development of digital health platforms, and the integration of behavioral intervention modules within maternal and child health systems, enabling more precise and effective prenatal health promotion. Future research should further explore how to optimize behavioral goal setting, provide personalized support, and reinforce triggering mechanisms. It is essential to offer structured physical activity guidance to all overweight or obese women before pregnancy to help increase their engagement in moderate-intensity physical activity.

## 5 Conclusions

The results of this study demonstrate that an FBM-based intervention significantly improved physical activity among pregnant women with pre-pregnancy overweight or obesity, particularly in controlling gestational weight gain and enhancing adherence to recommended physical activity levels. Future research should further optimize intervention designs by integrating various behavior change theories and exploring more precise and personalized approaches to promote sustained physical activity behaviors and improved health management throughout pregnancy.

Nevertheless, this study has certain limitations. First, all participants were recruited from a single tertiary general hospital located in Henan Province, China. The geographic scope of the sample was relatively narrow, and the study did not adequately capture potential variations across regions with different economic conditions, sociocultural contexts, health literacy levels, and healthcare resource allocations. This regional concentration may limit the generalizability and applicability of the intervention to other populations or settings, thereby constraining the broader external validity of the study findings. Second, although the intervention spanned the entire duration of pregnancy, some outcome indicators in the third trimester showed diminishing between-group differences, suggesting that future interventions should place greater emphasis on adjusting the pacing of intervention delivery and strengthening individualized support mechanisms in late pregnancy.

Therefore, future research should continue to optimize FBM-based intervention strategies while integrating other behavior change theories—such as Self-Determination Theory and the Theory of Planned Behavior—to develop multidimensional, personalized intervention models. It is also recommended that future studies adopt multi-center, cross-regional designs to broaden the participant base and improve the representativeness and generalizability of findings. Such approaches would allow for a more comprehensive evaluation of the applicability, effectiveness, and sustainability of FBM-guided interventions across diverse populations of pregnant women.

## Data Availability

The raw data supporting the conclusions of this article will be made available by the authors, without undue reservation.

## References

[1] Bull FC Al-Ansari SS Biddle S Borodulin K Buman MP Cardon G World health organization 2020 guidelines on physical activity and sedentary behaviour. Br J Sports Med. (2020) 54(24):1451–62. 10.1136/bjsports-2020-102955 33239350 PMC7719906

[2] Downs DS Chasan-Taber L Evenson KR Leiferman J Yeo S. Physical activity and pregnancy: past and present evidence and future recommendations. Res Q Exerc Sport. (2012) 83(4):485–502. 10.1080/02701367.2012.10599138 23367811 PMC3563105

[3] Zhang Y. Investigation and Analysis of Physical Activity Status Among Urban Pregnant Women in Tianjin and the Effect of Community-Based Exercise Intervention. Tianjin: Tianjin Medical University (2019).

[4] Nascimento SL Surita FG Cecatti JG. Physical exercise during pregnancy: a systematic review. Curr Opin Obstet Gynecol. (2012) 24(6):387–94. 10.1097/GCO.0b013e328359f131 23014142

[5] Liu XM Zhou TS Fan C Wang JL. Interpretation and insights of the 2019 Canadian guideline for physical activity during pregnancy. Sports Sci Res. (2020) 41(03):98–103. 10.12064/ssr.20200314

[6] Wang L Zhou B Zhao Z Yang L Zhang M Jiang Y Body-mass index and obesity in urban and rural China: findings from consecutive nationally representative surveys during 2004–18. Lancet. (2021) 398(10294):53–63. 10.1016/S0140-6736(21)00798-4 34217401 PMC7617101

[7] Fang HY Zhao LY Ju LH Guo HJ Jia FM Yu WT Analysis of malnutrition and overweight/obesity among women of childbearing age (15–49 years) in China. Chinese J Public Health. (2018) 34(09):1229–32. 10.11847/zgggws1116987

[8] Sun X. Analysis of the impact of combined lifestyle interventions on pregnancy outcomes in overweight and obese pregnant women. Chinese J Pract Village Doctors. (2022) 29(03):37–9. 10.3969/j.issn.1672-7185.2022.03.012

[9] Smith R Shakespeare J Williams Z Knight M Foster C Physical activity for pregnant women: an infographic for healthcare professionals. Br J Gen Pract. (2017) 67(663):460. 10.3399/bjgp17X692801 28963415 PMC5604810

[10] McGee lD Cignetti CA Sutton A Harper L Dubose C Gould S. Exercise during pregnancy: obstetricians’ beliefs and recommendations compared to American congress of obstetricians and Gynecologists’ 2015 guidelines. Cureus. (2018) 10(8):e3204. 10.7759/cureus.3204 30410829 PMC6207175

[11] Jácome C Almeida R Pereira AM Amaral R Mendes S Alves-Correia M Feasibility and acceptability of an asthma app to monitor medication adherence: mixed methods study. JMIR Mhealth Uhealth. (2021) 9(5):e26442. 10.2196/26442 34032576 PMC8188323

[12] Tan Z Jiang X. Research on interactive design of online learning platforms based on the Fogg Behavior Model (FBM). Pack Eng. (2020) 41(04):189–94. 10.19554/j.cnki.1001-3563.2020.04.024

[13] Yan R. Research on the Design of Interactive Toys for the Elderly Under the Urban “Family and Friends Mutual Assistance” Elderly Care Model. Xi'an: Xi'an University of Technology (2021).

[14] Hong YC Qian XB. Interactive design strategies for smart washing machines based on the Fogg Behavior Model. Design. (2024) 37(19):128–31. 10.20055/j.cnki.1003-0069.002185

[15] Wu G Gong J. Investigating the intention of purchasing private pension scheme based on an integrated FBM-UTAUT model: the case of China. Front Psychol. (2023) 14:1136351. 10.3389/fpsyg.2023.1136351 36968747 PMC10033583

[16] Sittig S Wang J lyengar S Myneni S Franklin A. Incorporating behavioral trigger messages into a mobile health app for chronic disease management: randomized clinical feasibility trial in diabetes. JMIR Mhealth Uhealth. (2020) 8(3):e15927. 10.2196/15927 32175908 PMC7105932

[17] Lu XX Zhu XQ Xu DN Zhang MY. Advances in the application of the Fogg Behavior Model in the field of health management. J Nurs Sci. (2025) 40(06):125–9. 10.3870/j.issn.1001-4152.2025.06.125

[18] Yang C. The Impact of a Medication Reminder Application Based on the Fogg Behavior Model on Antihypertensive Medication Adherence in Patients with Chronic Kidney Disease. Xinjiang: Xinjiang Medical University (2023).

[19] Agha S Bernard D Francis S Fareed A Nsofor I. Determinants of human papillomavirus vaccine acceptance among caregivers in Nigeria: a Fogg Behavior Model-based approach. Vaccines (Basel). (2024) 12(1):84. 10.3390/vaccines12010084 38250897 PMC10820200

[20] Sporrel K Wang S Ettema D Nibbeling N Krose B Deutekom M Just-in-time prompts for running, walking, and performing strength exercises in the built environment: 4-week randomized feasibility study. JMIR Form Res. (2022) 6(8):e35268. 10.2196/35268 35916693 PMC9379785

[21] Katirayi L Tchendjou P Tchounga B Mbunka M Wicks M Conserve D. Changing attitudes towards HIV testing and treatment among three generations of men in Cameroon: a qualitative analysis using the Fogg Behavior Model. BMC Public Health. (2023) 23(1):501. 10.1186/s12889-023-15139-3 36922812 PMC10015680

[22] Agha S Tollefson D Paul S Green D Babigumira J. Use of the Fogg Behavior Model to assess the impact of a social marketing campaign on condom use in Pakistan. J Health Commun. (2019) 24(3):284–92. 10.1080/10810730.2019.1597952 30945612

[23] Agha S Morgan B Archer H Babigumira J Guthrie B. Understanding how social norms affect modern contraceptive use. BMC Public Health. (2021) 21(1):1061. 10.1186/s12889-021-11110-2 34088295 PMC8178889

[24] Militello L Melnyk BM Hekler EB Small L Jacobson D. Automated behavioral text messaging and face-to-face intervention for parents of overweight or obese preschool children: results from a pilot study. JMIR Mhealth Uhealth. (2016) 4(1):e21. 10.2196/mhealth.4398 26976387 PMC4810011

[25] Fan JH. Design of Attention-Enhancing Products for Children with Autism Based on the Fogg Behavior Model (FBM). Qinhuangdao: Yanshan University (2022).

[26] Yang X Li H Zhao Q Han R Xiang Z Gao L. Clinical practice guidelines that address physical activity and exercise during pregnancy: a systematic review. J Midwifery Womens Health. (2022) 67(1):53–68. 10.1111/jmwh.13286 34841649

[27] Maxwell C Gaudet L Cassir G Nowik C McLeod NL Jacob C. Guideline no. 391-pregnancy and maternal obesity part 1: pre-conception and prenatal care. J Obstet Gynaecol Can. (2019) 41(11):1623–40. 10.1016/j.jogc.2019.03.026 31640864

[28] Borg GA. Psychophysical bases of perceived exertion. Med Sci Sports Exerc. (1982) 14(5):377–81. 10.1249/00005768-198205000-00012 7154893

[29] Woltmann ML Foster C Porcari JP Camic CL Dodge C Haible S. Evidence that the talk test can be used to regulate exercise intensity. J Strength Cond Res. (2015) 29(5):1248–54. 10.1519/JSC.0000000000000811 25536539

[30] Zhang Y Zhao Y Dong SW Xiong Y Hu XQ. Reliability and validity evaluation of the Chinese version of the pregnancy physical activity questionnaire. Chinese J Nurs. (2013) 48(09):825–7. 10.3761/i.issn.0254-1769.2013.09.019

[31] Yang HM Deng YF Gao LL. Reliability and validity of the Chinese version of the pregnancy exercise self-efficacy scale. Chinese J Nurs. (2017) 52(05):632–6. 10.3761/i.issn.0254-1769.2017.05.028

[32] Yao SS Duan XQ Zhang L Wang JM Liu XH. Survey on knowledge, attitudes, and practices regarding physical activity during pregnancy among pregnant women. Chinese J Fam Plan. (2018) 26(07):559–62. 10.3969/iissn.1004-8189.2018.07.004

[33] Johansson K Bodnar LM Stephansson O Abrams B Hutcheon JA. Safety of low weight gain or weight loss in pregnancies with class 1, 2, and 3 obesity: a population-based cohort study. Lancet. (2024) 403(10435):1472–81. 10.1016/S0140-6736(24)00255-1 38555927 PMC11097195

[34] Sui Z Grivell RM Dodd JM. Antenatal exercise to improve outcomes in overweight or obese women: a systematic review. Acta Obstet Gynecol Scand. (2012) 91(5):538–45. 10.1111/j.1600-0412.2012.01357.x 22229625

[35] Flannery C McHugh S Anaba AE Clifford E O'Riordan M Kenny LC. Enablers and barriers to physical activity in overweight and obese pregnant women: an analysis informed by the theoretical domains framework and COM-B model. BMC Pregnancy Childbirth. (2018) 18(1):178. 10.1186/s12884-018-1816-z 29783933 PMC5963099

[36] Marino KR Donnelly G Moore IS Vivo MD Vishnubala D. Pregnancy and physical activity: facilitating change. Br J Sports Med. (2023) 57(20):1285–6. 10.1136/bjsports-2023-107265 37507198

[37] Wang X Bai Y Li W Yang C Zhang L Zhu H Effect of artificial intelligence driven therapeutic lifestyle changes (AI-TLC) intervention on health behavior and health among obesity pregnant women in China: a randomized controlled trial protocol. Front Public Health. (2025) 13:1580060. 10.3389/fpubh.2025.1580060 40421363 PMC12104060

[38] Zhao S. Application of a Breastfeeding Intervention Based on Self-Efficacy Theory in Patients with Gestational Diabetes Mellitus. Qingdao: Qingdao University (2022).

[39] Heslehurst N Russell S Brandon H Johnston C Summerbell C Rankin J. Women’s perspectives are required to inform the development of maternal obesity services: a qualitative study of obese pregnant women’s experiences. Health Expect. (2015) 18(5):969–81. 10.1111/hex.12070 23617245 PMC5060880

[40] Wang C Wei Y Zhang X Zhang Y Xu Q Sun Y. A randomized clinical trial of exercise during pregnancy to prevent gestational diabetes mellitus and improve pregnancy outcome in overweight and obese pregnant women. Am J Obstet Gynecol. (2017) 216(4):340–51. 10.1016/j.ajog.2017.01.037 28161306

[41] Nobles C Marcus BH Stanek ER Braun B Whitcomb BW Solomon CG Effect of an exercise intervention on gestational diabetes mellitus: a randomized controlled trial. Obstet Gynecol. (2015) 125(5):1195–204. 10.1097/AOG.0000000000000738 25932848 PMC4418021

[42] Van der Ploeg HP Bull FC. Invest in physical activity to protect and promote health: the 2020 WHO guidelines on physical activity and sedentary behaviour. Int J Behav Nutr Phys Act. (2020) 17(1):145. 10.1186/s12966-020-01051-1 33239047 PMC7688396

[43] Mottola ME Davenport MH Ruchat SM Davies GA Poitras VJ Gray CE 2019 Canadian guideline for physical activity throughout pregnancy. Br J Sports Med. (2018) 52(21):1339–46. 10.1136/bjsports-2018-100056 30337460

